# Children’s affective involvement in early word learning

**DOI:** 10.1038/s41598-023-34049-3

**Published:** 2023-05-05

**Authors:** Vivien Outters, Robert Hepach, Tanya Behne, Nivedita Mani

**Affiliations:** 1 Department for Psychology of Language, University of Goettingen, Göttingen, Germany; 2 Leibniz Science Campus “Primate Cognition”, Göttingen, Germany; 3 Department of Experimental Psychology, University of Oxford, Oxford, UK; 4 Department of Developmental Psychology, University of Goettingen, Göttingen, Germany

**Keywords:** Psychology, Human behaviour

## Abstract

The current study set out to examine the underlying physiological mechanisms of and the emotional response associated with word learning success in young 3-year-old predominantly white children. In particular, we examined whether children’s physiological arousal following a word learning task predicts their word learning success and whether successful learning in turn predicts children’s subsequent positive emotions. We presented children (*n* = 50) with a cross-situational word learning task and measured their pupillary arousal following completion of the task, as well as changes to their upper body posture following completion of the task, as indices of children’s emotions following task completion. Children who showed greater physiological arousal following the novel word recognition task (*n* = 40) showed improved subsequent word recognition performance. We found that children showed more elevated posture after completing a familiar word learning task compared to completing a novel word learning task (*n* = 33) but results on children’s individual learning success and postural elevation were mixed. We discuss the findings with regards to children’s affective involvement in word learning.

## Introduction

Language is acquired in social settings, and exposure to linguistic input from caregivers and peers in social interactions is critical to language learning success. While there is undoubtedly an influence of input quality and quantity on language learning success (see^1^, for a review), children also actively shape their learning experience by selectively sampling from the information presented to them. Curiosity-driven or active learning approaches^2,3^ highlight the role of children who, like little scientists, choose when and what to learn from whom^1^. Such approaches highlight our natural disposition to learn more about our environment, due to the resolution of curiosity being intrinsically rewarding^4^ and linked to more positive affective states and hippocampal dopamine release^5^. Against this background, the current study examines the affective states associated with word learning success in young children. First, we examine the extent to which the internal state of the child, with regard to their physiological arousal during and after a word learning task, predicts successful learning of novel word-object associations. Furthermore, we examine whether successful learning predicts the subsequent internal state of the child with regard to positive emotions that may be triggered by their success at the task.

### Curiosity driven learning

Curiosity may be defined as a state of interest or arousal triggered by input that is potentially novel, deviant from one’s expectations or, in some cases, just beyond one’s repertoire of knowledge^2,3,6^. It is often intertwined with discussions of intrinsic motivation^7–9^, which may be operationalized as a drive to participate in an activity or acquire knowledge due to the pleasure that is derived from doing so, rather than due to any external motivation triggered by extrinsic (e.g., material) rewards (although see work by^10^ that the distinction between intrinsic and extrinsic rewards can be notoriously blurry). Historically, theorists initially tied curiosity to the novelty of a stimulus, with^6^ suggesting that curiosity was increased when people experience an intermediate state of familiarity with a newly presented stimulus. Thus, if the stimulus is too far removed from what one knows, there is reduced conflict between the presented information and one’s representation of the world, leading to reduced curiosity. The same is the case if the newly presented information overlaps considerably with concepts already familiar to the learner. Echoes of this suggestion remain in “knowledge gap” theories, which suggest that curiosity is triggered by a need to reduce uncertainty in one’s knowledge about a particular topic^11^.

Empirical support for such suggestions is provided by studies reporting an inverted-U-shape in participants’ curiosity with regard to their knowledge about a certain topic. For instance, in one study, participants reported being maximally curious about answers to questions they knew something about, while being less curious about questions they knew nothing or too much about^12^. Participants’ curiosity was predicted by the pupillary response prior to the answer being revealed, with more dilated pupils in participants who reported being more curious about the answer to a question^12^. Increased curiosity also predicts retrieval of information, with participants better recalling the answer to a question they were more curious about (^4,6^; see^13^ for results with a large scale database and review). Indeed, here, even stimuli that were ‘accidentally’ presented in a high curiosity phase were retrieved with greater success relative to stimuli presented in a low curiosity phase^4^. Similar findings are reported in studies examining neurocomputational models of learning, with maximal learning success reported when the model is allowed to select the stimuli in a curious manner, i.e., by maximizing the novelty of the stimuli to be presented with regard to the model’s prior experience with that stimulus.

Curiosity-driven learning in early childhood is less well researched, but the evidence suggests that learning is similarly motivated by children’s interest in particular stimuli in their environment. These studies suggest that, from early on, infants actively direct their caregivers to provide them with information that they are interested in^14,15^ and also retain such information better (^16,17^ but see^18^). Children also selectively (and retrospectively) prioritize information from instructors they have reason to believe are more knowledgeable relative to less knowledgeable instructors^19,20^ and choose when they want further information from their instructors, actively eliciting clarifying information in more referentially ambiguous contexts^21^. Finally, they are also selective with regards to the complexity of the stimuli they attend to, focusing, for instance, on stimuli that are neither too complex or too simple^22^. Thus, children play an active role with regards to what they want to learn, when they want to learn and whom they want to learn from.

Theories examining the role of intrinsic motivation on learning success have typically focused on our affective state during learning, i.e., the extent to which we are engaged in a task, as well as the affective state associated with learning success, i.e., the emotions associated with having learned something successfully (e.g., the learning progress hypothesis^8^). Thus, according to this hypothesis, we actively seek out information that improves our internal representations of the world around us, thereby being most interested or engaged with stimuli that optimally increase our knowledge. This is based off studies suggesting that our internal state of arousal during a task or motivation to complete a task is associated with learning success (in school-aged children^23,24^, adults^12^), and that the positive emotions associated with having completed a task are similarly associated with learning success^25,26^. Furthermore, neuroimaging research has recently examined the link between learning and the reward circuitry in the brain. The increasing number of studies suggesting the involvement of the ventral striatum in reward processing^4,27^, and findings that the level of activity in the striatum is linked to participants’ success at word learning^28^ and their interest in learning^4,12^, see^2^ for a review), point to an intrinsic component to participants’ motivation to learn. Developmental accounts further increasingly speculate that this possibility may also apply to language acquisition in early development^1,29^ and that the pace of early word learning may in part be explained by the notion that word learning success in children is associated with positive emotions. For instance^29^ suggest that hearing the label of a presented stimulus and inferring the intention of the speaker may be a high value inference for children in referential communication.

Thus, one of (many) factors driving early word-object association learning is that word-learning may generate positive emotions, potentially associated with reward, because children acquire information relevant for future referential communications with each new learned word-object association. Against this background, we examine the extent to which children’s state of arousal during and following completion of a task predicts word learning success in children, and furthermore, the extent to which word learning success results in positive emotions.

#### Word learning and recognition

Word learning in young children can be assessed by presenting children with images of as yet unfamiliar objects and training them on the labels for these objects. Following such training, children are then presented with two (or sometimes four) images of the previously trained objects and hear the label for one of these objects. Increased fixations to the labelled objects following presentation of the label for this object are typically interpreted as evidence for children’s learning success and recognition of the trained word-object associations (see^30^ for a review of early word learning research in young children^18,31,32^. In the cross-situational variant of the word learning task employed here^33^, children are trained on temporarily ambiguous word-object mappings, where ambiguity can be resolved when children consider the information presented across the entire training session. Here, children are presented with two unfamiliar images in training and hear two different novel labels, without being told which object is referred to by which of the two labels. In subsequent trials, however, the labels and objects presented are controlled so that every time children see a particular object, they hear the label for this object, so that they can use the frequency with which a particular label co-occurs with a particular object to infer the correct word-object mappings. At test, children are then presented with two of the trained objects and hear the label for one of these objects. As noted above, increased fixations to the labelled object are interpreted as evidence of children having learned the word-object mappings. Children’s eye-movements can, therefore, provide us with further information of their learning of novel word-object mappings in such tasks.

#### Pupil dilation

The human pupil dilates in response not just to the physical properties of a stimulus but also indexes mental and emotional processing load (see^34^ for a review of the literature in the 60 decisive years of pupillometry research). Following initial suggestions of pupillary changes reflecting changes in arousal state^35^ subsequent studies documented increased pupil dilation during states of cognitive load^36^. Based on their review of the research on pupillometry, Laeng and colleagues further suggest that the pupil may offer “a window to the preconscious”, by indexing changes in participants’ allocation of attention and engagement^37–39^, motivation and sustained processing^40–42^, and how rewarding or important a particular stimulus might be^18,43,44^. Some of these findings have been replicated with developmental populations. For instance, Hepach and colleagues recorded children’s tonic pupil dilation, i.e., a sustained change in pupil diameter over a longer period, during presentation of neutral videos as an index of their emotional arousal in response to previously presented emotionally valent stimuli^40,41^, and suggest that increases in pupil dilation can reflect children’s motivation to help others attain their instrumental goal^42^. With regards to the current study, the findings briefly reviewed suggest that the pupillary response during a task may reliably index participants’ arousal during and after a task, potentially even when participants do not display awareness or sensitivity to the stimuli being presented^45,46^. Therefore, it is possible that stimuli which are of high intrinsic value (reward) to children will result in greater physiological arousal, i.e., pupil dilation, (see e.g.^47^) which may in turn relate to how well children learn new words (see^18^).

#### Body posture

Studies examining changes in participants’ body posture have suggested a link between a person’s emotional state and their body posture (see^48^ for a review). These studies report an association between a person’s feeling of shame or pride with their upper body posture, with a more upright body posture and erected head indexing pride and a lower body posture with lowered head indexing shame^49,50^. Similarly, success at a difficult task or failure at an easy task have been shown to induce changes in children’s body posture at three years of age^49^, highlighting consistency in the findings across the life span^42^. show that 2-year-old children display increased positive emotions, indicated by more upright body posture, when they successfully achieve a goal for themselves or when they successfully help someone else achieve a goal—but posture is lower if no goal is achieved for anyone (see^51,52^ for similar applications of the measure). The authors particularly stress the link between body posture and emotional expression and highlight the possibility that task success could trigger increased positive emotions which is reflected in elevated body posture. The latter suggestion is based on their findings of an increase in adult chest height following a positive emotion, relative to a negative emotion^48,53^. Corroborating evidence in these tasks is delivered by behavioral coding. Manual coding of the valence of the participants, e.g., how happy the child looked and how much they smiled, correlates with children’s increase in body posture, providing additional evidence for the use of body posture as an index of participants’ emotional state^54^. Against this background, the current study will examine the extent to which word learning success is associated with changes to children’s upper body posture as an index of the positive emotions that may be triggered by successful word learning. This would mirror previously documented increases in posture when children complete goals for themselves and others^42^.

### The current study

The current study examined the association between children’s internal affective state during and following completion of a word learning task and their word learning success. Children have been shown to learn novel word-object associations from a very early age (see^30^, for a review). Here, we specifically employed a potentially more difficult cross-situational word learning task^33^ given our interest in the degree of variability in word learning success. In this task, three-year-olds are presented with six novel word-object associations, where the one-to-one mappings between words and objects can be inferred based on the frequency of co-occurrence of a particular label with an object across trials. Children are then tested on their learning of the word-object associations in a subsequent test phase. Here, we presented them with images of two novel objects side-by-side on the screen and the label for one of these images, and examined their fixations to the labelled image as an index of learning success. We examined children’s internal state of arousal (indexed by pupillary arousal) during learning and at test and were particularly interested in the latter as an index of how engaged they were in the task. Furthermore, we also examined the extent to which word learning success itself (indexed by their eye-movements during the cross-situational word learning task) may be associated with children’s level of engagement following the task (pupillary arousal) and the positive emotions (indexed by changes in body posture) experienced by children following successful recognition performance.

We measured children’s looking behaviour, pupil dilation, and body posture at different points in the study. We expected that children would show increased fixations to the labelled object during test as an index of their learning and recognition of the trained word-object associations.

We measured children’s change in pupil dilation before, during, and after the training phase but our analyses predominantly focussed on the measure taken before and after testing children’s recognition of the newly learned word-object associations as well as on the pupil dilation measure taken before and after testing their recognition of the familiar word-object associations. Pupillary changes from before to after testing children’s recognition of the newly learned word-object associations was our index of children’s emotional state of arousal following task completion^18^. We hypothesized that children who showed greater pupil dilation following the novel word recognition test phase would show better recognition of the novel word-object associations, highlighting increased arousal related to task success. Thus, we anticipated that children who were better able to perform in the task would also show increased arousal following task completion. Furthermore, while the focus of the current study was on pupillary arousal during task performance, as an index of their engagement in the task, we also hypothesized that children who showed greater pupillary arousal during the learning phase would show improved learning of the word-object associations.

Changes in children’s body posture were measured at several points during the session, namely, at the beginning of the study, after training of the novel word-object associations, as well as after testing their recognition of the newly learned word-object associations, and finally, after testing their recognition of a series of familiar word-object associations. Changes in body posture from before to after word recognition test was our critical index of children’s emotional state following task completion. We hypothesized that children who showed greater word learning success, i.e., improved recognition of the novel word-object associations, would experience more positive emotions due to their success at the task, which would be indexed by elevation of upper body posture following task completion.

## Results

### Word recognition

**Figure 1:**
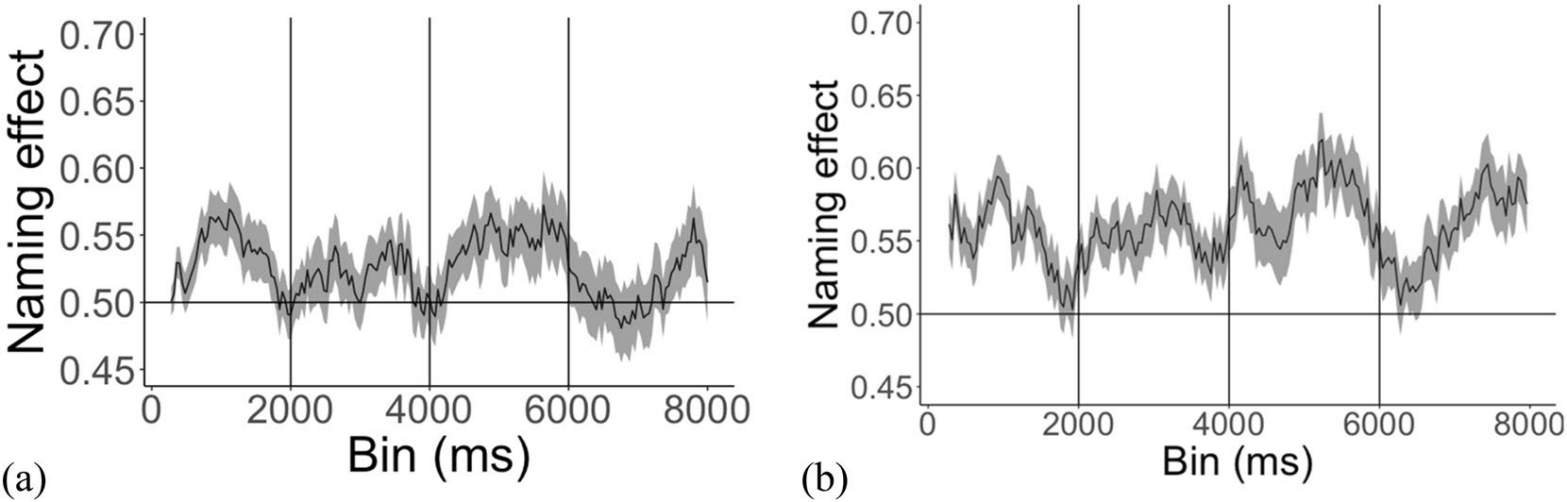
Proportion of target looking (SE in grey) during the novel word recognition test phase (**a**) and the familiar word recognition test phase (**b**).

Children learned the novel word-object associations and recognized the familiar word-object associations presented, i.e., showed increased fixations to the labelled object relative to the other distracter object in the novel and familiar word recognition test trials. One sample t-tests, comparing the PTL to chance (50%), found that the proportion of fixations to target was above chance in both the novel word recognition, *t*(46) = 3.44, *p* = 0.001 95% CI 0.51, 0.55 (see Fig. 1a), and familiar word recognition phases, *t*(43) = 8.41, *p* < 0.001, 95% CI 0.57, 0.61 (see Fig. 1b).

### Word recognition and upper body posture

**Figure 2:**
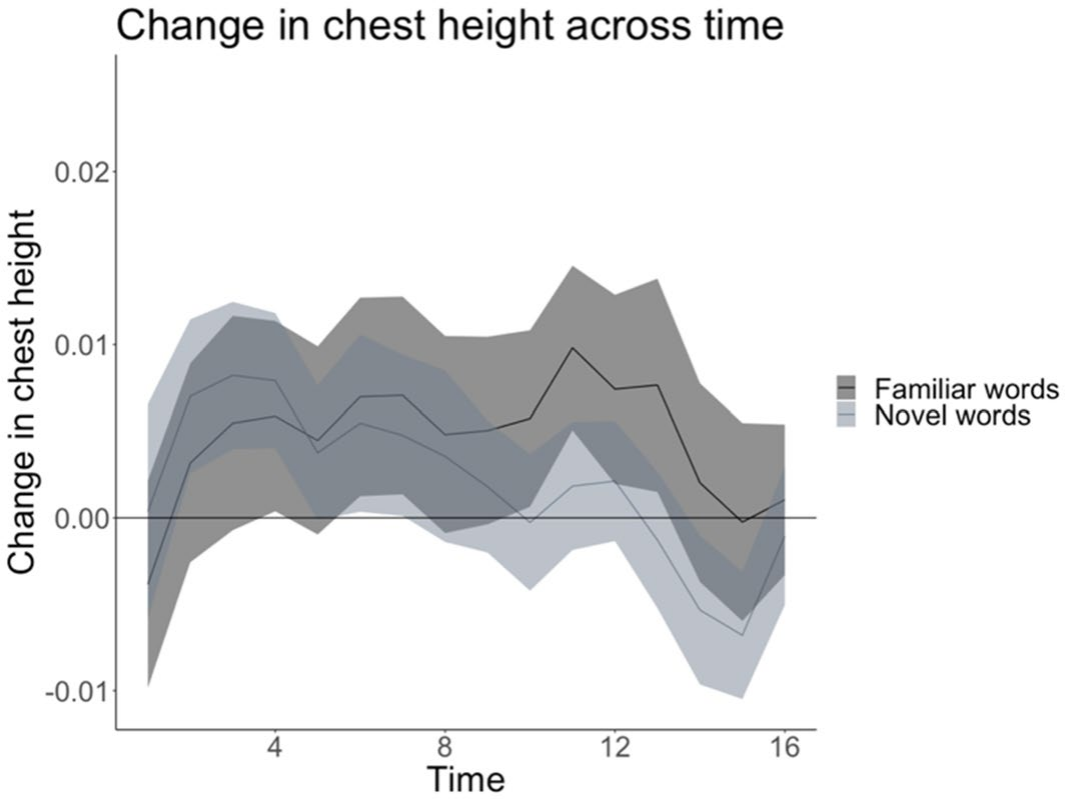
Changes in upper body posture (chest height) over time separated in novel word recognition test phase and familiar word recognition test phase.

We fitted a full model (as described in the “Methods”) and compared this to a reduced model excluding the factor *Word recognition* (PTL) and found that adding *Word recognition* and the interaction between this factor and *Time* and *Familiarity* (novel word recognition, familiar word recognition) improved model fit, *χ*^2^ = 36.84, *df* = 4, *p* < 0.001. Table 1 presents the model parameters and output for the data on upper body posture (chest height) and Word recognition (PTL). As Table 1 suggests, we found an interaction between *Time:Familiarity:Word recognition*, suggesting differences in the change in upper body posture across novel word recognition and familiar word recognition trials, which differed according children’s word recognition success in the two phases. Figure 2 plots the change in children’s upper body posture across novel and familiar word recognition phases and suggests, in keeping with the results presented in Table 1, that children had higher upper body posture following familiar word recognition trials relative to novel word recognition trials.

**Table 1:** Model examining the upper body posture data including *Time* and its interaction with *Word recognition* (*proportional target looks*) and *Familiarity* (*novel words, familiar words*), as well as fixed effects of *Age* (in days) and *Gender* and random intercepts for subject and random slope for time (lmer(upper body posture ~ Time × Word recognition × Familiarity + (1 |gender) + (1 |age.ds) + (1 + Time|id)).

Predictors	Estimates	SE	*t*	*p*
Intercept	0.002	0.004	0.610	0.541
Time	0.0003	0.0003	1.037	0.299
Word recognition	0.042	0.037	1.149	0.250
Familiarity	0.007	0.003	2.215	0.026
Time: word recognition	− 0.011	0.003	− 2.959	0.003
Time: familiarity	− 0.001	0.0003	− 3.262	0.001
Word recognition: familiarity	0.030	0.048	0.615	0.538
Time: word recognition: familiarity	0.011	0.004	2.298	0.021

The second, focused, analysis revealed significant 2-way interactions of *Time* and *Familiarity* (*χ*^2^ = 11.03, *df* = 1, *p* < 0.001), *Word recognition* and *Familiarity* (*χ*^2^ = 27.998, *df* = 1, *p* < 0.001), but not for *Time* and *Word recognition* (*χ*^2^ = 3.69, *df* = 1, *p* = 0.055). With regards to the interaction of *Time* and *Familiarity*, further exploratory analyses revealed that children’s chest height remained elevated after the familiar word recognition task (*beta* = 0.001, *SE* = 0.002, see Table 2) while posture decreased after novel word recognition task (*beta* = − 0.004, *SE* = 0.002, see Table 3). With regards to the interaction of *Word recognition* and *Familiarity,* children who looked longer at the correct novel label during the test phase had higher postural elevation after novel word recognition task (*beta* = 0.006, *SE* = 0.001, see Table 3) whereas we found the converse pattern for children’s recognition of the familiar label, (*beta* = − 0.004, *SE* = 0.001, see Table 2).

**Table 2:** Model examining the upper body posture data using focussed two-way interactions referenced to the *familiar* word recognition phase: upper body posture ~ (z.Time + Familiarity + Word recognition)^2^ + (1| gender) + (1|age) + (1 + z.Time||id). Differences in the interactions across Tables 2 and 3 reflect merely the reference level.

Predictors	Estimates	SE	*t*	*p*
Intercept	0.006	0.003	1.947	0.051
Time	0.001	0.002	0.632	0.527
Word recognition	−0.004	0.001	−2.788	0.005
Familiarity	−0.002	0.002	−1.288	0.197
Time: word recognition	−0.002	0.001	−1.926	0.054
Time: familiarity	−0.005	0.002	−3.334	< 0.001
Word recognition: familiarity	0.009	0.001	5.331	< 0.001

**Table 3:** Model examining the upper body posture data using focussed two-way interactions referenced to the *novel* word recognition phase: upper body posture ~ (z.Time + Familiarity + Word recognition)^2^ + (1| gender) + (1|age) + (1 + z.Time||id). Differences in the interactions across Tables 2 and 3 reflect merely the reference level.

Predictors	Estimates	SE	*t*	*p*
Intercept	0.004	0.003	1.285	0.199
Time	− 0.004	0.002	− 2.528	0.001
Word recognition	0.006	0.001	− 2.788	0.005
Familiarity	0.002	0.002	1.288	0.197
Time: word recognition	− 0.002	0.001	− 1.926	0.054
Time: familiarity	0.005	0.002	3.334	< 0.001
Word recognition: familiarity	− 0.009	0.001	− 5.331	< 0.001

For all the above-reported analyses on children’s upper body posture, we found similar patterns of results with regards to the association between word recognition and lower body posture (see Appendix A).

**Figure 3:**
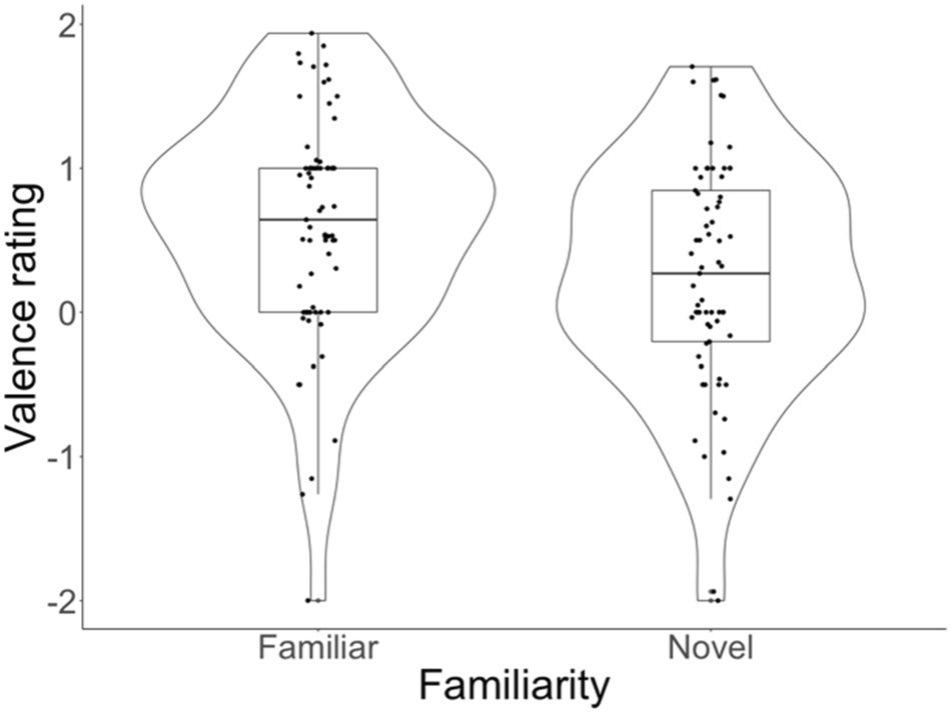
Difference in valence rating between novel word recognition test phase and familiar word recognition test phase.

Next, we examined coders’ ratings of children’s emotional valence based on emotional face cues. A linear mixed effects model including *Word recognition* and its interaction with *Familiarity* found an influence of *Familiarity*, suggesting that children were rated as experiencing more pleasant, positive emotions following the familiar word recognition phase relative to the novel word recognition phase (see Table 4 and Fig. 3) which corroborates the results on body posture changes.

**Table 4:** Model examining the valence data including *Word recognition* and its interaction with *Familiarity (novel word recognition, familiar word recognition)*, as well as random intercepts of Age (in days), Gender, Subject and Coder: lmer(valence measure ~ Word recognition × Familiarity + (1 |gender) + (1 |age) + (1||id) + (1|coder)).

Predictors	Estimate	SE	t	p
Intercept	0.581	0.142	4.076	< 0.001
Word recognition	− 0.019	1.200	− 0.016	0.987
Familiarity	− 0.407	0.104	− 3.913	< 0.001
Word recognition: familiarity	− 2.344	1.514	− 1.547	0.121

### Word recognition and pupil dilation

#### Training phase

Spearman’s correlations investigating the relation between pupillary arousal during the training phase (difference between before to mid training and before to after training) and performance during the novel word recognition test phase showed no correlation between pupillary arousal during training and performance at test (*ps* > 0.05).

#### Novel word recognition test phase

**Figure 4:**
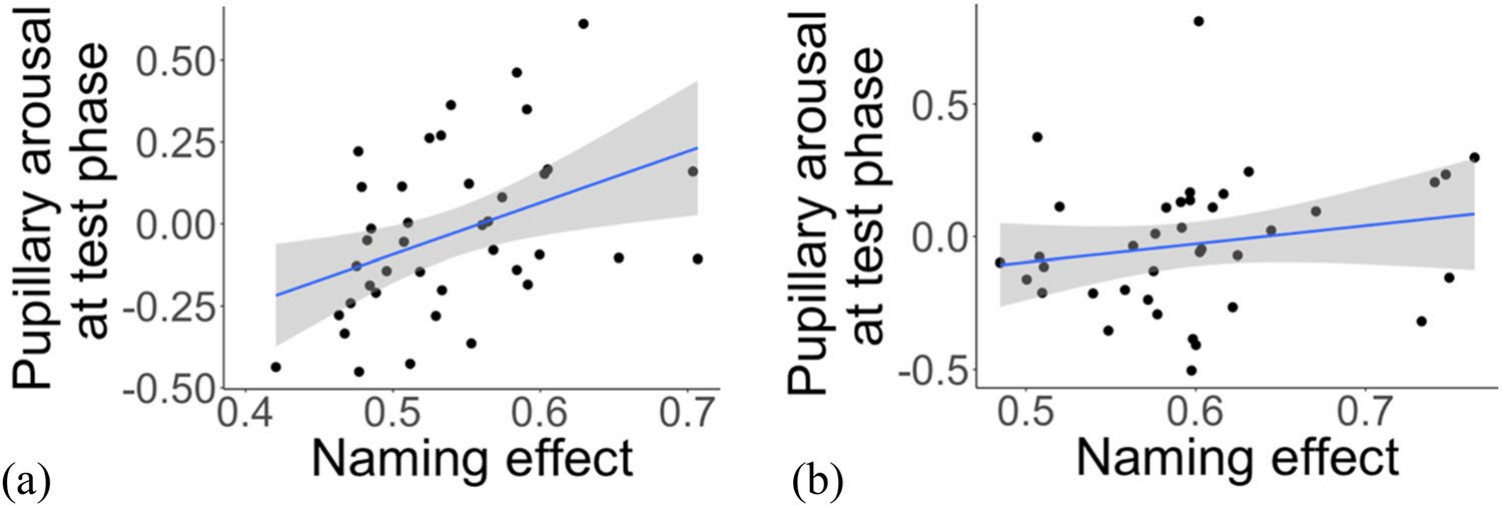
Correlation between pupillary arousal during the test phase and word recognition in the (**a**) novel word recognition and (**b**) familiar word recognition test phases.

A Spearman’s correlation investigating the relationship between the change in pupillary arousal following the test phase (difference in pupil dilation from before to after the novel word recognition test phase) and word recognition success (PTL) revealed a positive correlation, *r*(38) = 0.41, *p* = 0.009 (see Fig. 4).

#### Familiar word recognition test phase

A Spearman’s correlation investigating the relationship between the change in pupillary arousal following the test phase (difference in pupil dilation from before to after the novel word recognition test phase) and word recognition success (PTL) found no correlation between pupillary arousal during test and performance at test, *r*(36) = 0.19, *p* = 0.24.

## Discussion

Against the background of suggestions that learning, in adults, may be rewarding in and of itself, i.e., that we may be intrinsically motivated to learn^4,12,28^ the current study set out to examine the relationship between successful word learning in children and the emotional arousal and valence triggered by word learning, indexed by pupillary arousal and upper body posture, respectively. In particular, we hypothesized a positive relationship between performance in the novel word recognition task and pupillary arousal upon task completion, indexing the relationship between task engagement and task success (cf.^18^), as well as a positive relationship between word learning success and upper body posture following task completion, indexing positive emotions related to task success (see^42^ for similar findings on children’s instrumental task completion).

### Word recognition

We found that children showed successful recognition of both the trained word-object associations that they were introduced to in the cross-situational word learning paradigm as well as the familiar word-object associations. In both cases, children looked more at the image of the labelled object relative to the unlabeled distractor object. Since objects were counterbalanced so that they were targets as often as they were distractors, increased looking at an object when presented with the label for this object can be reasonably interpreted as evidence for novel word-object association learning and recognition, as well as familiar word recognition.

### Word recognition and pupil dilation

We examined changes in pupillary arousal during task completion with regards to the training phase, the novel word recognition test phase and the familiar word recognition test phase. We found no evidence for an association between pupillary arousal during the training phase and the novel word recognition task. Neither did we find evidence for an association between pupillary arousal during the familiar word recognition test phase and performance in the familiar word recognition task. However, we did find positive evidence of an association between increase in pupillary dilation during completion of the novel word recognition task and word recognition success in this task.

As noted earlier, the human pupil dilates in response to a range of cognitive processes including attention and resource allocation, memory load, cognitive load as well as emotional factors such as motivation and reward, engagement and perseverance. In particular, a number of studies report an association between pupil size and interest or engagement in a task^18,34,55^. For instance, pupillary arousal to musical excerpts has been found to vary with participants’ engagement in music in their daily lives or their interests in particular kinds of music^34,55^ suggesting associations between long-term individual interests and pupillary responses to particular types of stimuli. Similarly, pupillary arousal to categories of familiar objects has been shown to predict children’s learning of novel word-object associations for objects from categories individual participants showed increased arousal to^16^. Pupillary arousal has also been shown to predict participants’ prosocial behaviour, with participants who showed increased pupillary arousal also being more willing to help those in need^39^, potentially therefore investing more in the situation.

Against this background, the positive association between pupillary arousal during the novel word recognition task and performance in this task could be explained by suggesting that children who invested more cognitive resources or effort during the task showed improved recognition of the novel word-object associations. In other words, those children who were more engaged or aroused by the task showed improved recognition performance (see^56^ for similar results with adults; and^57^ arguing for pupil dilation as an index of effort exertion). Admittedly, the directionality of this association is difficult to ascertain. For instance, participants could be more aroused or engaged in a task due to their being able to complete the task or they may perform better in the task because they are more engaged in the task. We will, therefore, steer clear of attributing directionality either way and highlight merely our interpretation of the association between pupillary arousal and task performance as indexing the association between increased, attention, engagement or effort allocation and improved task performance.

It is important to distinguish between resource or effort allocation during encoding and during recognition, since we did not find any evidence for an association between pupillary arousal during the training phase and novel word recognition performance. Such a finding could have been interpreted as evidence for improved attentional allocation during learning leading to improved encoding and subsequent recall of the word-object associations at test, i.e., increased learning success. Given the reported null effect in this analysis, we will leave open the question regarding the association between resource/effort allocation and encoding.

Furthermore, we only found an association between pupillary arousal and performance in the novel word recognition test but not the familiar word recognition test. One explanation for our failure to find evidence for such an association in the familiar word recognition task might tap into the ease of this task—thus, there may have been fewer demands on resources or less of a need to engage further in the task in order to show successful performance in this task. In other words, children may have been able to complete this task without engaging too much in the task or investing greater cognitive resources in the task, thereby allowing for less variation in pupil size during task completion. In contrast, the novel word recognition task may have called for greater allocation of resources leading to the reported association between pupil size and task performance in this task.

Finally, we highlight one additional interpretation of the reported increase in pupil size related to task performance that is more in keeping with the aims of the current study. In particular, there has been some suggestion that increase in pupil size may be related to emotional factors such as motivation and reward. In particular,^43^ suggest that pupillary dilation may be increased for more (monetarily) rewarding tasks but only in cases where the task requires additional effort. While this interpretation is closely tied to participants’ motivation or engagement to complete a task (as discussed above), it adds a further perspective in terms of the association between how rewarding and how demanding a task is. Thus, our finding of the association between increased pupillary arousal and performance in the novel word recognition task but not the familiar word recognition task, could potentially be interpreted as tapping into this nexus between reward and task difficulty, with the former being difficult enough to engage participants in the task and being potentially more rewarding.

### Word recognition and body posture

We hypothesized that children who showed improved word recognition performance would also show increased upper body posture following task completion as an index of the positive emotions generated by successful task completion. This was motivated by studies showing children’s and adult’s success at a task is similarly associated with their emotional state and more upright body posture (see^48^ for a review;^42,49^). Thus, for instance, children display increased positive emotions, indicated by more upright body posture, when they successfully achieve a goal or when they successfully help someone else achieve a goal. Importantly, it is not children’s elevated posture per se that reflected positive emotions but rather the direction of the effects was such that positive outcomes, which are generally associated with positive emotions, resulted in elevated body posture. In contrast, negative events such as failing to achieve a goal^42,58^, receiving undeserving rewards^51,59^, or making unjustified requests for help^52^ resulted in lowered body posture.

However, we found mixed evidence that those children who showed improved task performance showed more upright body posture. Overall, children showed more elevated posture after they completed the familiar word recognition task suggesting that ‘easy’ tasks resulted in positive emotions. In contrast, children’s posture decreased after completing the novel word recognition task, which could be interpreted as suggesting that, following more 'difficult’ tasks, children’s emotions are less positive. Indeed, following the novel word recognition phase, children’s positive emotions (postural elevation) were contingent on children’s individual word learning success as indexed by their respective PTL-scores. The fact that we did not find a similar positive relation between children’s recognition success and posture changes following the familiar word recognition phase could be attributed to the fact the children had overall high recognition success. The present study is the first to relate individual differences in children’s posture changes to another outcome variable and the mixed nature of the present individual differences analyses suggest that the depth-sensor-imaging technique to capture posture changes is more sensitive to group-level effects (on children’s emotional valence). The measures of pupil dilation (arousal) and body posture (emotional state) allowed us to capture the underlying mechanisms and resulting positive emotions of successful word learning. We consider this a first step toward addressing the question whether word learning is intrinsically rewarding. To further probe the reward mechanisms underlying children’s acquisition of new words, at least two avenues for future research are possible. First, if word learning is intrinsically motivated (rewarding) then external rewards should undermine the motivation to learn words. One could conceive of an experimental paradigm similar to the classic overjustification effect studies^9^ where children are given materials rewards for learning new words. Assuming that learning is intrinsically rewarding, postural elevation should be lower, even after learning a novel word, compared to a condition in which no reward is given. A second avenue would be to look at the predictive value of children’s change in posture in relation to their future learning success. If the measures of body posture reflect positive emotions from successful learning then the greater the increase in posture after learning success, the better, or at least more perseverant, learning should be on future trials.

Importantly, our finding that children were reported as experiencing more pleasant or positive emotions and showed elevated upper body posture following familiar word recognition relative to novel word recognition highlights the validity of the posture measure in tapping into children’s affective state. While this result was independent of the performance of the children during the word recognition test phases, these findings suggest that children were potentially happier after performing an easier task, as indexed by the posture measure and validated by the valence coding. We explain this finding by suggesting that their increased positive affect following familiar word recognition may have been due to the ease of the task, although we note that it may have also been caused by other factors such as their familiarity with the stimuli presented in the familiar word recognition test. Taken together, we suggest that the posture measure employed here appears to be able to tap into children’s affective states during the study but was not, however, associated with children’s word learning success.

On the one hand, our finding that children’s body posture was unrelated to word recognition performance appears to contradict with the findings on body posture reviewed above, as well as suggestions of the connectivity between motivational and learning circuits in the brain^60,61^. On the other hand, the lack of a significant association can owe to a number of factors which we briefly discuss here. For instance, studies highlighting the link between learning and motivation and/or reward circuity in adults have typically tapped into more implicit measures of reward, e.g., increased fMRI activation in the ventral striatum in cases of successful learning^28,60^. Thus, it is possible that our measure of body posture and its relation to participants’ positive emotional state may not adequately tap into how rewarding word learning may be for young children. Alternatively, it is possible that children may not be as aware of the fact that they have learned the words in the current task as they may be of other aspects of their behavior. For instance, this body posture measure has been shown to index positive emotions upon successful goal completion in other tasks, e.g., in helping others^48^. The contrast between these findings might suggest that children may not be as aware of their successful word learning relative to their having helped someone, leading to increased positive emotions following such prosocial behaviour. Thus, awareness of having learned a word requires a degree of metalinguistic awareness that may be particularly difficult, especially for such young children in such an implicit task. Future studies may, therefore, want to consider making learning success more explicit to the child, e.g., by having them identify the correct object more explicitly via pointing, in order to tap into the link between learning success and positive emotions. Finally, we note that children may have been aware of having successfully learned words, but any positive emotions that may have been generated as a result of this may be more explicit at other times during the experiment. Thus, children may have experienced positive emotions at some other point during the task, e.g., when they successfully recognized the words relative to the point at which we examined changes in body posture as our chosen index of such positive emotions. Thus, considerably more work is needed to further investigate the relationship between positive emotions, expressed in changes in body posture, and word learning success.

The current study set out to examine the role of children’s internal affective state on their word learning success. In keeping with active learning approaches that highlight the contribution of the child to learning success, we found the children who were more engaged in or aroused by the task showed improved word recognition performance. While we found no evidence of greater level of engagement during encoding being associated with word recognition performance, the correlation between level of engagement at test and performance at test, highlights the important role of children’s motivation and engagement in performance in such tasks. Notably, we did not find any evidence that children experienced positive emotions upon successfully learning words, at least as indexed by changes to their upper body posture. While not entirely in keeping with the literature on the affective state of word learning in adults, we suggest a number of reasons why children may be affectively involved in word learning, as well as highlighting further avenues for future research.

## Methods

### Participants

Participants were recruited from the lab infant and child database, which caregivers of young children can register to in order to participate in our studies. Participants were typically developing monolingual children with no vision, hearing or mobility issues. Data were collected in the laboratory of the corresponding author. Fifty monolingual German children between 36 to 48 month of age (average 3 years; 2 months; 28 days; range 3 years, 0 month, 4 days to 3 years, 11 months, 30 days, 29 girls) were recruited for this study. One child was excluded from the study due to experimental error and a further child was excluded due to their knowing one of the novel objects prior to the study. Thus, there were 48 children in the final sample, but not all children provided data for both measures. Forty-seven children provided eye movement and pupillometry data given that data collection failed at an early stage in the task for one child. Forty children provided body posture data. No data could be recorded for the other eight children due to technical problems. Finally, 39 children provided data for both eye tracking and posture measures. Parents gave informed written consent for their child’s participation in the study and children received a book in appreciation for their participation. Ethics approval was given by the Psychology Institute’s Ethics committee. All research was performed in accordance with the relevant guidelines and regulations.

### Stimuli

**Figure 5:**
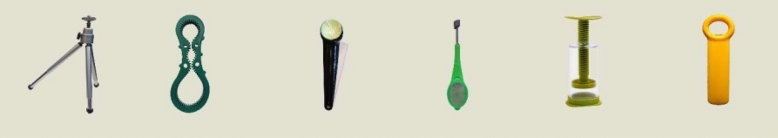
Unknown objects presented during the study.

Children were trained on six novel word-object associations. The chosen novel words were *akan, basa, sibu, modi, isot, upos*. The novel objects were six objects of roughly the same size which were assumed to be unfamiliar to the children (see Fig. 5). Parents were asked after the experiment whether any of the objects were familiar to the child. A female native German speaker was recorded saying the words in isolation in infant directed speech. During the training phase, a single token of two different novel words were spliced together such that one of the novel words was presented 500 ms into the trial, and a different novel word was presented 500 ms after the end of the first word. This compiled auditory stimulus was then merged with still images of two of the novel objects, presented to the left and right side of the screen with images measuring approximately 680 and 890 pixels separated by a distance of 40 pixels. During the test phase, individual tokens of the novel words were spliced together to create separate auditory stimuli for each novel word, where, participants heard four repetitions of the same word at 0 ms, 2000 ms, 4000 ms and 6000 ms into the trial, e.g., *akan, akan, akan, akan*. The compiled auditory track was merged with still images of two of the novel objects (used during training), one of which was the named target object, presented to the left and right side of the screen.

A separate set of stimuli was used for the familiar word recognition test phase. Here, we presented children with pairs of images of familiar objects (*dog*, *duck*, *ball*, *frog,* and *car*) as they heard the labels for one of these objects repeated four times (as in the novel word recognition test phase). For details of counterbalancing of labels and objects across all phases, refer to the description of the procedure below.

### Procedure and design

**Figure 6:**
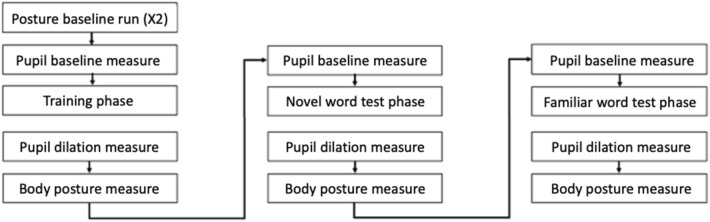
Order of study phases.

We recorded both eye tracking data (eye movements and pupillary measures) as well as body posture data (change in body posture) at different points during the study (Fig. 6). For the eye tracking task, eye movements and pupil diameter were captured using a Tobii eye tracker (X120) with a gaze and pupil diameter sampling rate of 120 Hz. The eye tracking data were collected in a quiet room with only artificial lighting. The eye tracker was placed on a platform underneath the TV screen (1920 × 1080 pixel) on which the stimuli were presented. The child sat in a car seat about 65 cm away from the monitor. Body posture was recorded using a Kinect motion capture camera (Microsoft Kinect) connected to a separate laptop. The Kinect camera was placed on a small table next to a toy, in a room adjacent to the room where testing took place. Before the experiment started, the child was shown where the toy was and was told that they would be asked to walk to this toy multiple times during the study. The child walked a distance of approximately 5 m between the door to the testing room and the toy. The caregiver was told to keep their distance from the child so as not to be tracked during measurement of the child’s body posture (details on the recording pipeline available here: https://github.com/rhepach/Kinect).

The eye tracking study was split into three phases, namely a training phase, a novel word recognition test phase, and a familiar recognition test phase (see Fig. 6). The child provided eye-movement data during each of the phases. Pupillary data was acquired before and after the training, novel word test phase and familiar word test phase, as well as in the middle of the training phase (not shown in the Figure for simplicity). Body posture data was acquired before the learning phase as well as before and after the training, novel word test and familiar word test phase.

Once the child felt comfortable with the experimenters, one experimenter and the child walked to the testing room where the child received a building block. The child was told to walk to the adjacent room and place the building block in a toy located directly next to the Kinect camera, which the experimenter had previously pointed to. This procedure was performed twice during the baseline run capture (X2 in Fig. 2 above). The child was told to walk to the toy again at the end of the training phase, after completion of the novel word recognition test phase, and at the end of the experiment, i.e., after completion of the familiar word recognition test phase.

Following the baseline walks, the child was escorted to the testing room and seated in a child-seat in front of a TV screen, with the eye-tracker placed directly underneath the TV screen. Calibration was conducted using a 5-point calibration procedure. Once calibration was completed, we recorded the first pupillary measure of children’s baseline tonic pupillary response as they watched a 10 s video of bubbles floating around the screen accompanied by chimes^40,48,62^. This neutral video was repeated in the middle (after 15 training trials) and at the end of the training phase, at the beginning and end of the novel word recognition test phase and at the beginning and end of the familiar word recognition test phase, allowing us to measure children’s pupil dilation (as an index of arousal) at critical points throughout the experiment.

#### Training phase

The training phase presented children with 30 trials, where each trial consisted of two of the novel objects presented to the left and right side of the screen respectively as the child heard the labels for each of these objects repeated once. The objects were presented in silence for 500 ms, followed by the onset of the label for one of the objects. The onset of the label for the other object was 500 ms after the offset of first label. Each trial lasted four seconds. Thus, while children could not infer the respective word-object associations from a single trial (since they saw two objects and heard two labels in each trial), across trials, they would hear the label for a particular object every time they saw this object onscreen. Children could use the co-occurrence of label and object across trials to infer the intended word-object pairings. Across the experiment, each object was labelled 10 times. The pairing of objects across trials was counterbalanced such that each object appeared 10 times, five times to the left and five times to the right of the screen, paired with each of the other five objects. The order of the words in each trial was counterbalanced such that each word appeared 10 times (accompanied by the object it was representing), being mentioned first and second in the trial an equal number of times. Importantly, we ensured that there was no correlation between the side of presentation of the object and the position of the word in the trial. Thus, if the word was presented first in the trial, the object could be either on the right or on the left side of the screen, depending on the trial. A fixation cross appeared between each trial to ensure that the child was looking at the screen before the next trial started. Counterbalancing of the assignment of labels to particular objects resulted in six different lists and participants were randomly assigned to one of the lists. Thus across the lists, each of the objects was associated with each of the labels. The entire training phase lasted five minutes. Fifteen trials into the training phase, children were presented with the neutral bubble video to allow measurement of their pupillary dilation halfway through the experiment. We similarly presented children with the bubble video at the end of the training phase to measure their pupillary dilation at the end of the training phase. Then, children were given a building block and told to walk by themselves to the toy in the next room, to allow measurement of their body posture.

#### Novel word recognition test phase

After they had played with the toy for a short while (approximately 2–3 min), children were asked to return to the testing room. They were seated in front of the eye tracker and presented with the bubble video again to allow measurement of their pupillary dilation before the onset of the novel word recognition test phase. Then, children were presented with 12 trials. In each trial, children saw two of the previously presented novel objects to the left and right side of the screen for eight seconds and heard the label for one of these objects repeated four times at 0, 2000, 4000 and 6000 ms into the trial, with the entire trial lasting 8000 ms. Across trials, each object appeared four times, half of the time as the target and the other half of the time as a distractor, and half of the time to the left and the other half of the time to the right side of the screen. The bubble video was presented at the end of the novel word recognition test phase, allowing us to measure children’s pupil dilation at the end of the novel word recognition test phase. Next, children were given a building block and asked to walk to the toy in the adjacent room, allowing us to measure their body posture at the end of the novel word recognition test phase.

#### Familiar word recognition test phase

After a brief period playing with the toy and the building block, children were asked to return to the testing room and seated on the chair. The bubble video was presented once more allowing us to measure children’s pupil dilation at the beginning of the familiar word recognition test phase. Then, children were presented with five familiar word recognition trials where they saw two familiar objects and heard the label for one of the objects onscreen. The label was presented at 0, 2000, 4000, 6000 ms into the trial, with the entire trial lasting 8000 ms. Each of the five known objects (dog, duck ball, frog, and car) appeared twice across the entire phase, once as the target object and once as the distractor object, i.e., children heard the label for this object in one trial and heard the label for the other object in the other trial. The bubble video was presented one last time to allow for the measurement of children’s pupil arousal at the end of the familiar word recognition phase. The children then received a building block for the last time and were asked to walk to the toy in the adjacent room, allowing us to measure their body posture at the end of the familiar word recognition test phase.

The children were then allowed to play for a while with the toy before receiving a book in appreciation for their participation in the experiment while the caregiver was informed about the purpose of the study.

### Data analysis

For each child, we examined their word learning and recognition, pupillary dilation, and body posture across different phases of the experiment.

#### Word recognition

The eye tracking output from Tobii during the novel and familiar word recognition test phases was extracted and further analysed in combination with the stimulus information for each trial. The eye tracker provides an estimate of where children were looking every 8 ms. Only data which were coded as reliable, i.e., with a validity less than 2, were included in the analyses. These data were further aggregated across 40-ms bins, with each of these 40-ms bins coded for whether the child was looking at the target or the distractor. Based on the location and size of the objects on the left and right side of the screen, we defined areas of interest and coded these for whether they were the target or a distractor in the trial. Data from a particular trial were only included and further analyzed if we obtained data for at least 20% of the trial (see^1^) leading to an exclusion of 46 trials (7.73%) during the novel word recognition test phase and 1 trial (0.12%) in the familiar word recognition test phase. We required that participants provided data for at least two trials in each test phase to be included in the analyses. This led to no exclusions. We calculated the proportion of target looking (PTL) for each trial based on the total amount of time children spent looking at the target over the amount of time children spent looking at the target and the distractor across the entire trial. This measure was then aggregated across trials for each child, resulting in one mean PTL value over all trials for each child. We entered this PTL value into further analyses described below. Forty-seven children provided data for the novel word recognition test phase, while 44 children provided data for the familiar word recognition test phase (two children did not return to the eye tracker booth for the familiar word recognition test phase and one child did not provide enough data during this phase).

#### Pupil dilation

Pupillary data collected during presentation of the bubble video at the beginning and end of the training phase, the novel word recognition test phase and the familiar word recognition test phase were extracted from the Tobii output. Pupil diameter was sampled at 120 Hz. Data from both eyes were extracted for each time bin. Time bins were only included in the analysis if they were part of a fixation and valid eye tracking data (average over both eyes less than 2 on a Tobii scale). Data from a trial were only included if we had data for at least 20% of that trial. Data were then filtered by removing data from timepoints where the difference in pupil size between adjacent time points was in the upper 10% of the difference in pupil size between all adjacent time points were removed^16^. This ensured that large differences in pupil size between two adjacent samples, which are likely to be artifacts, are excluded from further analysis. Missing data were interpolated across every four samples, equivalent to a 70 ms sliding window^40^. Filtered, interpolated data from each eye were then averaged together, if data from both eyes were provided, or using only the data from one eye when this was not the case. For the novel word recognition test phase, 40 participants provided data both before and after the onset of the test phase (one child did not provide any data for the novel word test phase, six children did not provide data on both measurements, before and after the novel word test phase). For the familiar word recognition, 43 children provided data both before and after the familiar word recognition test phase. We aggregated the pupillary data across the entire duration of the bubble video and subtracted the pupillary measure before the onset of the training phase and each test phase from the measure obtained after completion of each phase respectively. This difference score was then entered into subsequent analyses (described below).

#### Body posture

Data were preprocessed using a separate MATLAB script. In a first step, we filtered and cleaned the data to ensure that we did not include skeletons of adults and we only included data (1) that were in our tracking range (3.2 m–1.2 m distance from the Kinect camera), (2) where the skeleton point center back was above the skeleton point hip center, (3) the skeleton point of the head was above the skeleton points of the shoulder, (4) the skeleton point center hip was between the skeleton points left and right hip, (5) the skeleton point center shoulder is between the skeleton points right and left shoulder. These criteria ensured that only datapoints (frames) that could reasonably be considered skeletal measurements of the child were included in the analyses (see^42^). Resulting gaps in skeletal measurements were then interpolated. Following these criteria, we excluded an additional 6 participants. Two did not provide valid data for baseline walks (for one participant only the adult was tracked, one participant turned back in the middle of the walks), three participants whose data violated the checks outlined above. We further excluded the data from one participant who incorrectly received the test phase before the learning phase. An additional eleven single walks were removed (child walking backwards (1), child walking wrong way (2), child turned back (3), child fell or grabbed something on the ground (3), child and adult tracked as one skeleton (1), and child was walking with big toy (1)). We focused on two body points in particular, namely the y-coordinate (height) of the chest’s center to estimate the upper body posture change, and the y-coordinate of the hip center to estimate lower body posture change^42^. We scaled the posture measurements over time, such that all participants provided data for 20 data points in each run, regardless of how long they spent on each run. Thus, we divided the run into twenty time bins and extracted an average posture measure for each of these time bins. The posture measure is typically less accurate the closer the participant is to the Kinect camera. To ensure that these less accurately measured data points are excluded from the analysis, we only considered the data for the first 16 time bins collected (c.f.^54^).

Next, we baseline corrected the data acquired after completion of the novel word recognition test or familiar word recognition test phase. During baseline correction, we subtracted the baseline posture data from the measure obtained after completion of each phase for each time bin and each phase separately. If the child provided data for both baseline walks, baseline correction was performed using the average of both walks. If the child did not provide data for the first baseline walk, the second baseline walk was taken for the baseline correction. This baseline corrected difference score was then entered into subsequent analyses (described below).

### Analysis

#### Word recognition

First, we examined whether children had learned the novel word-object associations and recognized the familiar word-object associations presented. Here, we ran two one-sample t-tests to examine whether the PTL was above chance (50%) in the novel word recognition and familiar word recognition phases, separately.

#### Word recognition and body posture

To examine the relationship between word recognition and body posture, we examined the data from 34 children who provided both body posture data and eye movement data from both the novel word recognition test phase and the familiar word recognition test phase. We ran linear mixed models on the baseline-corrected chest-height (reflecting changes in upper-body posture) and hip-height (reflecting changes in lower-body posture). The first model included *Time* and its interaction with *Word recognition* and *Familiarity*, as well as random intercepts of *Age* (in days, z-transformed), *Gender* and *Subject*, and random the slope for *Time*. *Time* referred to the 16 time bins in each walk, where the data in each bin for the critical run was baseline corrected to corresponding data in each bin in the baseline run (or runs depending on whether the child provided data for a single or both baseline runs). *Word recognition* was the PTL-score from the novel word recognition test phase or the familiar word recognition test phase, depending on which phase was being analysed, indicated by *Familiarity (Novel word recognition, Familiar word recognition phase)*. Since our focus was on whether word learning success was associated with increased body posture, we compared this full model to a null model excluding *Word recognition*. Breaking this model down according to *Familiarity*, we ran a model on the novel word recognition test phase and the familiar word recognition test phase separately. Following this omnibus test of our predictor variables we fitted a second model which was similar to the initial model but included all possible two-way interaction of *Familiarity, Time,* and *Word recognition.* As stated above, the dependent measure was either the change in the children’s chest height or hip height (reported in the Appendix) across separate models.

We collected additional data on the valence of the child’s emotion, provided by two coders. The two adult coders were blind to the study’s hypotheses and phases. They rated the emotions of the child on an individual trial level, based on frame-by-frame recordings of each trial. Each coder was asked to rate how pleasant the emotion was, the child was experiencing (most positive = 3, least positive = 1^38^). We calculated the mean rating for each phase of the study (baseline, training, novel word recognition and familiar word recognition) and calculated the difference between baseline and all other phases, to receive a value expressing the change in the children’s affective state during the experiment (positive value = more positive emotion compared to baseline, negative value = less positive emotion compared to baseline). We merged the two coders’ values and ran a linear mixed model on the valence value including *Word recognition* and its interaction with *Familiarity,* as well as random intercepts of *Age* (in days, z-transformed), *Gender*, *Subject,* and *Coder*.

#### Word recognition and pupil dilation

To examine the relationship between word recognition and pupillary arousal, we examined the data from 40 children who provided both pupillary data (for the training and the novel word recognition test phase) and eye movement data from the novel word recognition test phase, and 38 children who provided both pupillary data and eye movement data from the familiar word recognition test phase. We ran Spearman’s correlations on the pupil dilation and gaze data. The pupil measure used in the analysis was the difference in pupil dilation from before the test phase to after this phase, using either the data collected from the novel or familiar word recognition test phase, depending on the phase being analyzed across separate correlations. The PTL-measure was the PTL-score for each trial from the novel word recognition test phase or the familiar word recognition test phase, across separate correlations. Additionally, we ran Spearman’s correlations on the pupil dilation data during the learning phase and PTL data during the novel word recognition phase. The pupil dilation measure was the difference in pupil dilation from before to mid and after learning.

## Supplementary Information

Supplementary Information.

## Data Availability

All data, analysis code, and research materials are available at https://osf.io/27e6v/?view_only=86964c1df4314ddb8dcb0f9a934a5f69. Data were analyzed using R version 4.1.2 (R Core Team, 2020).
