# Supplementary material for: Children’s affective involvement in early word learning
Source: Sci Rep. 2023 May 5;13:7351. doi: 10.1038/s41598-023-34049-3 (PMC10162962; doi:10.1038/s41598-023-34049-3)
Supplement: Supplementary file 1 — Supplementary Information. [file 41598_2023_34049_MOESM1_ESM.docx]

**Appendix**

***Word recognition and lower body posture.*** Table 1 presents the model parameters and output for the data on lower body posture (hip height) and word recognition (PTL). As Table 1 suggests, we found the same pattern of results for hip height (lower body posture) as was reported with chest height (upper body posture, reported above). There was a change in posture across trial which differs across the two conditions with higher lower body posture following familiar word recognition relative to novel word recognition. Lower body posture drops across time following novel word recognition (see Figure A1)

*Table 1.* Model examining the lower posture data including *Time* and its interaction with *Word recognition* and *Familiarity (novel words, familiar words)*, as well as fixed effects of *Age* (in days) and *Gender* and random intercepts for subject and random slope for *Time*: lmer(lower body posture ~ Time*Word recognition*Familiarity + (1 |gender) + (1 |age.ds) + (1+time|id))

| Predictors | Estimate | SE | t | p |
| --- | --- | --- | --- | --- |
| Intercept | 0.005 | 0.007 | 0.834 | .404 |
| Time | 0.0007 | 0.0005 | 1.1485 | .137 |
| Word recognition | 0.058 | 0.048 | 1.189 | .234 |
| Familiarity | 0.008 | 0.004 | 1.935 | .053 |
| Time: Word recognition | -0.014 | 0.005 | -2.88 | **.004** |
| Time: Familiarity | -0.001 | 0.0004 | -3.793 | **<.001** |
| Word recognition: Familiarity | 0.105 | 0.063 | -1.671 | .094 |
| Time :Word recognition: Familiarity | 0.021 | 0.006 | 3.298 | **<.001** |


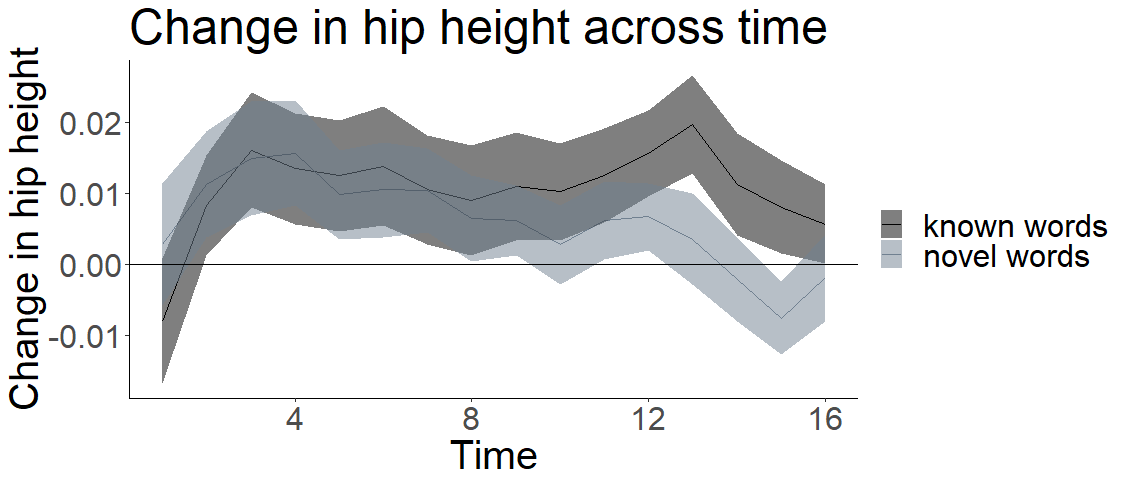


*Figure A1.* Changes in lower body posture (chest height) over time separated in novel word recognition test phase and known word recognition test phase.
